# Demographics of the Disappearing Bottlenose Dolphin in Argentina: A Common Species on Its Way Out?

**DOI:** 10.1371/journal.pone.0119182

**Published:** 2015-03-18

**Authors:** Els Vermeulen, Stefan Bräger

**Affiliations:** 1 Laboratory of Oceanology, MARE Research Centre, University of Liège, Liège, Belgium; 2 International Seabed Authority, Kingston, Jamaica; Biodiversity Research Center, Academia Sinica, TAIWAN

## Abstract

Populations of the once common bottlenose dolphin (*Tursiops truncatus*) in Argentina have precipitously declined throughout the country in the past decades. Unfortunately, local declines of common species are easily overlooked when establishing priorities for conservation. In this study, demographics of what may well be the last remaining resident population in the country were assessed using mark—recapture analysis (Pollock’s Robust Design) of a photo-identification dataset collected during 2006–2011 in Bahía San Antonio (Patagonia, Argentina). Total abundance, corrected for unmarked individuals, ranged from 40 (95%CI: 16.1–98.8) to 83 (95%CI = 45.8–151.8) individuals and showed a decrease over the years. Adult survival rates varied between 0.97 (± 0.037 SE) and 0.99 (± 0.010 SE). Average calving interval equalled 3.5 ± 1.03 years, with 3.5 births/year in the entire population and a minimum annual birth rate of 4.2%. However, data suggest that calves may have been born and lost before being documented, underestimating birth rate, calf mortality, and possibly the number of reproductive females. Either way, the recruitment rate of calves appears to be insufficient to support the size of the population. This population is relatively small and declining. Considering the disappearance of populations north and south of the study area, an incessant decline will have severe consequences for the continuous existence of this species in Argentina, indicating an urgent need for serious conservation efforts. This study provides insight into how the failure to recognize local population declines can threaten the national (and eventually the international) status of a common species like the bottlenose dolphin.

## Introduction

Coastal bottlenose dolphin (*Tursiops truncatus*) populations in Argentina have declined notably in the past decades, with sightings being extremely rare nowadays in regions where they were once common [1,2]. Unfortunately, these declines have been ignored continuously resulting in possibly a single resident population remaining in the country [3].

Currently, the bottlenose dolphin is red-listed as of “least concern” in Argentina, but also as “conservation dependent” [1]. Furthermore, the hunting and killing of this (and of all other marine mammal) species is prohibited under national law (Ley 25.577/02) as well as under the provincial law of Rio Negro (Ley 4115/2006). However, a national or provincial management or conservation plan for this species does not exist.

It occurs that threatened populations of common species are overlooked frequently when priorities for biodiversity conservation are established. However, the failure to recognise local population declines, and thus the failure to apply the necessary conservation measures, causes once-common species to slide towards extinction [4]. The common bottlenose dolphin provides an example for a species believed to be common and widespread with its global conservation status listed as of least concern [5], although an ever increasing number of coastal populations have been reported to be declining over the past decades and to be seriously threatened by human activities thus becoming endangered in many regions worldwide [6,7]. Such population declines cannot only affect the global status of the species, but will also have subsequent ecological effects given the general role of the bottlenose dolphin as an apex predator. Despite generally being known to be a resilient species [8], the reported causes of these declines are often related to either habitat degradation [9], prey depletion [10] or contamination [8]. The latter is of concern because of the increasing understanding of the wide ranging effects of pollutants on the health condition and reproduction of the species [11].

In this study, demographics of what may be the last remaining resident coastal population in Argentina were assessed using mark-recapture analysis (Pollock’s Robust Design) of a photo-identification dataset collected between 2006 and 2011 in Bahía San Antonio (Province of Río Negro). Although the species is known to be among the best studied cetacean species in the world, our results provide only the second robust estimates for bottlenose dolphins in the Southwest Atlantic [12], and are the first estimates for the species from Argentinian waters. Therefore, the obtained information is believed to be critical for any attempt to avoid the looming disappearance of the bottlenose dolphin along the coasts of Argentina, indicating the urgent need for serious conservation efforts. Furthermore, this study provides an example on how the failure to recognize local population declines can threaten the national (and eventually the international) status of a once common marine species.

## Materials and Methods

### Study area

**Fig 1:**
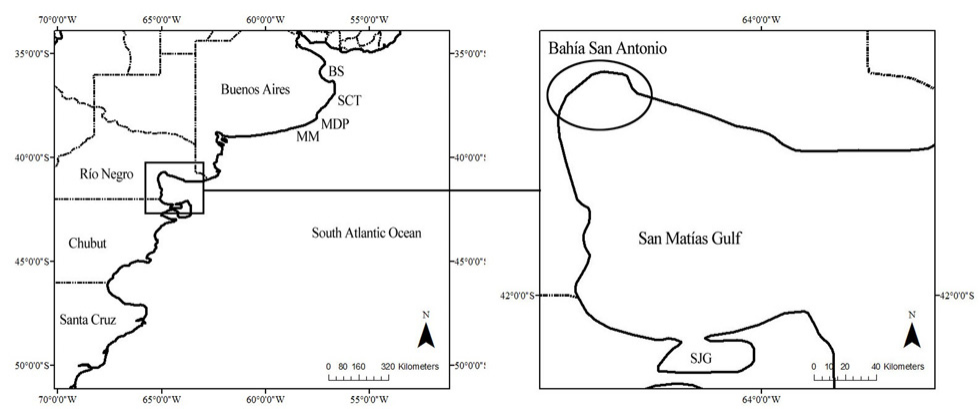
Map of Argentina indicating the location of the study area. Regions where bottlenose dolphins were studied between the 1970’s and 1980’s are also indicated (north to south: Bahía Samborombón (BS), San Clemente del Tuyú (SCT), Mar del Plata (MDP), Miramar (MM), San José Gulf (SJG)). The circle on the right indicates the location of the study area Bahía San Antonio

Bahía San Antonio (BSA, 40°45´S 64°54´W; Fig. 1) is a shallow bay of approx. 655 km² and a maximum depth of no more than 30 m, located to the northwest of the San Matías Gulf, Patagonia, Argentina.

### Survey effort and data collection

Between 2006 and 2011, 356 systematic photo-identification surveys of bottlenose dolphins were conducted, of which 227 surveys were land-based and 129 were conducted from a small outboard-powered inflatable boat. This effort totalled 1470 h during which 415 dolphin groups were observed. Additional photo-identification data were obtained opportunistically on 30 different occasions. Field permits were granted by the Secretaría de Ambiente y Desarrollo Sustentable and the Dirección de Fauna Silvestre of the Río Negro Province.

Seasons were defined as follows: (1) Summer: January-March, (2) Autumn: April-June, (3) Winter: July-September, (4) Spring: October-December (Table 1).

**Table 1:** Hours of photo-identification survey effort over the different seasons (in brackets the number of additional opportunistic photo-identification surveys).

	Summer	Autumn	Winter	Spring
**2006**	0	0	80.6	0
**2007**	26.6	174.6	123.1	42.2
**2008**	178.3	45.2 (1)	65.3 (4)	64.6 (3)
**2009**	87.0	120.5 (3)	48.8 (9)	17.6 (1)
**2010**	31.0	14.0 (3)	194.3 (2)	26.3
**2011**	77.9 (1)	32.6	19.6	0 (3)
**TOTAL**	**400.8**	**386.9**	**531.7**	**150.7**

A bottlenose dolphin group was defined as all individuals within a 100 m radius of each other, interacting or engaged in similar activities [13,14,15,16]. Each individual in the group was categorised as a neonate, calf, immature or adult. Neonates were defined by their small size (less than 1⁄3 the length of an adult), foetal folds [17], and close association with an adult [18]. Calves were categorised by being between 1/3 and 2⁄3 of the length of an adult, without foetal folds and mostly swimming in close association with an adult. Immatures were defined as individuals of similar size to an adult [19,20] but with a lighter coloration and an overall lack of severe scars and marks on their dorsal fins and flanks [21]. Furthermore, they were no longer in a close association with an adult. Adults were defined by their large size, darker coloration and higher number of permanent marks on the edge of their dorsal fins and flanks. Dolphins identified to be closely accompanied by a calf or neonate on at least two different occasions were assumed to be females [17, 22].

During each encounter, high-quality photographs of the dorsal fins of all individuals in the group were obtained, regardless of the obvious presence of clear marks, for later identification [21,23]. The survey of a given group was considered to be complete once photographs were obtained from all individuals in the group. The number of bottlenose dolphins and their age classes were verified later during photo-identification analysis.

### Photo-identification analysis

To ensure data quality, only good quality pictures were used in this analysis to avoid misidentifications, as poor-quality photographs are known to lead to biased estimates [24,25]. Individuals with no or few distinct marks were not used for any further analysis in this study. Immature individuals, calves and neonates were only used in the estimation of the proportion of well-marked individuals in the population. Since the acquisition of marks and scars on small cetaceans is cumulative over time [21], all distinctly marked individuals used in the analysis were assumed to be adults. Only photo-identification data from the years 2009 to 2011 were selected for the analysis of mark-recapture estimates of abundance and survival rates, whereas all photo-identification data gathered between 2006 and 2011 were used for the analysis of birth demographics.

### Modelling procedures

Mark-recapture histories were compiled and analysed using Pollock’s Robust Design [26,27,28,29] within the program MARK [30]. Data were structured in temporarily closed (i.e. without gain or loss due to immigration or emigration, birth or death) secondary sampling periods within primary periods that are separated by a longer time interval and assumed to be open.

The following parameters were estimated under the full-likelihood parameterization: apparent survival probability (*φ*) being the probability of surviving and staying in the study area (this is the sum of true survival and fidelity to the study area), abundance of marked individuals (*N*), the probability of temporary emigration (γ”) or being unavailable for capture given that the individual was available during the previous sampling occasion, the probability that an emigrated individual remained outside the study area or unavailable for capture during subsequent sampling (γ’) [28,29], and capture probability (*p*). The probability of recapture (*c*) was set to equal the capture probability (*p*) as photo-identification is known not to provoke a trap response (*p = c*).

From the closed and open population models [31,32], a set of models were considered; without time-dependent effect (.), with time-dependent effect between primary periods (*t*), with time-dependent effect within primary periods (*s*), with time-dependent effect between and within primary periods (t*s), with time-dependent effect over the different season (*season*), with time-dependent effect over the different years (*annual*), and with the combination of all these effects.

After selecting the most parsimonious model, three temporary emigration patterns were considered in the model set being (1) no temporary emigration (γ” = γ’ = 0) where there is no emigration at all, (2) random temporary emigration (γ” = γ’) where the probability of an individual being present in the study area is independent on whether or not it was present in the study area during the previous sampling period, and (3) Markovian temporary emigration (γ”γ’) where the probability of an individual being present in the study area is conditional on whether it was present in the study area during the previous sampling period or not [9,29,33,34]. The model with no emigration (γ” = γ’ = 0) was used as a basis to investigate the time-dependence of the estimated parameters. To explore the effects of heterogeneity in capture probabilities, additional models within Pollock’s Robust Design were fitted to the data using Pledger’s [35] mixture models, with a maximum of 2 mixtures. However, heterogeneity in capture probabilities has not been included in the models that incorporated temporary emigration as, according to [29], full-likelihood estimators have not yet been developed for these models.

### Model selection procedures

There is no goodness-of-fit (GOF) test available in MARK for Robust Designs [30]. However, in order to evaluate potential violations of assumptions inherent to capture-recapture data sets (see [34] for more information), the closed primary periods were used (by pooling all data of the secondary periods) within the Cormack-Jolly-Seber (CJS) framework to carry out adhoc GOF tests using the program Release. The variance inflation factor (median C-hat), being a measure of potential over- or under-dispersion, was then estimated in MARK in order to evaluate the need for a correction within the robust design. Where median C-hat was close to 1, the model with the lowest AICc (Akaike’s Information Criterion) value in the robust design was selected as the most parsimonious model [36]. Nevertheless, models within two AICc units have support from the data and should not be dismissed [37]. Therefore, final parameter estimates and respective SEs were averaged across all models in the candidate set based on the AICc weights, to account for model uncertainty [37].

Further, the Likelihood Ratio Test (LRT) was used to test specific biological hypotheses between nested models.

### Total abundance

As all well-marked individuals selected for the analysis were assumed to be adults [21], the obtained models only render estimates of survival rate and abundance for the adult portion of the population. Therefore, in order to estimate the total abundance (*N*_*total*_), the proportion of unmarked individuals (1- θ) (including immature individuals, calves and neonates) in the population was estimated by dividing the total number of unmarked individuals by the total number of individual dolphins observed per dolphin group [38,39]. This was achieved for all encounters where it was believed that all individual dolphins were photographed, and were averaged over all dolphin groups encountered within a primary period. The total abundance (*N*_*total*_) was then corrected by inflating *N* with the correction factor (1- θ). The standard error (SE) of the total abundance (*N*_*total*_) was calculated using the delta method [34] as:

$$SE\left(Ntotal\right)\;=\;\sqrt{Ntotal^{2}\left(\frac{SE\left(N\right)²}{N^{2}}+\frac{1-\theta }{n\theta }\right)}$$

where θ is the proportion of marked individuals in the population, 1- θ is the proportion of unmarked individuals in the population, and *n* is the total number of dolphin groups used to estimate θ. Log-normal 95% confidence intervals were calculated following [40], with a lower limit of *N* (low 95%CI) = *N*_total_/*C* and upper limit of *N* (up 95%CI) = *N*_total_ × *C*

$$C\;=\exp\begin{array}{c} \left(1.96\sqrt{\ln\left(1+{\left(\frac{SE\left(Ntotal\right)}{Ntotal}\right)}^{2}\right)}\right) \end{array}$$

### Birth demographics

Birth demographics were assessed from photo-identification data obtained between 2006 and 2011 from the 14 identified reproducing females with their closely associated calves. Calving intervals were assessed and averaged for all reproducing females. Deaths of calves were inferred from the abrupt disappearance of a calf from its mother’s side within the first 3 years of its life [41,42,43]. The birth season was estimated for each newly observed calf taking into account its size and the presence/absence of foetal folds, assuming foetal folds would be discernable up to an age of 6–8 months [17]. The minimum annual birth rate was estimated by dividing the average number of calves born per year by the estimated maximum population size [20].

### Population viability analysis

Based on the obtained population and reproductive parameters, a population viability analysis (PVA) [44] was performed using the program VORTEX [45], to evaluate the current trend in population persistence. When the required data was not available for our local population, published values were used from long-term studies on the species. A sensitivity analysis was then performed to evaluate the contribution of each parameter to the population growth rate.

## Results

### Photo-identification

The photo-identification catalogue includes 67 individuals of which 10 were assumed to be immature dolphins. The rate of first-time identifications diminished notably over the years, with no new adults identified in the final 2 years of the study, suggesting that all adult individuals in the population were identified by the end of 2009. The average number of re-identifications of identified individuals was 18 times (± 11.2 SD), ranging from 2 to 44 sighting days.

### Modelling procedure

The encounter histories of 45 individuals were used as a subset for the estimation of abundance and survival rates. Twelve primary periods were chosen within all 12 seasons of 2009, 2010 and 2011, with daylong survey trips within each season as secondary samples. The secondary samples were separated by short time periods to ensure closure of the population, whereas primary periods were separated by at least 1.5 months (Table 2).

**Table 2:** Duration of primary periods (consecutive days) and the number of secondary samples (survey trips) within each primary period used in Pollock’s Robust Design, and number of adult dolphins identified or re-identified within each primary period.

		Duration primary period (days)	Number of secondary samples (survey trips)	Individuals identified within the primary period
2009	Summer	29	7	35
	Autumn	24	6	14
	Winter	26	7	40
	Spring	9	3	28
2010	Summer	15	3	24
	Autumn	2	2	14
	Winter	22	12	38
	Spring	7	3	21
2011	Summer	8	3	23
	Autumn	9	4	10
	Winter	30	5	30
	Spring	20	3	5

The adhoc GOF tests did not indicate any lack of fit of the data (effect of transience Test 3.sr: p = 0.35; effect of capture on survival Test 3.sm: p = 0.97; test for recapture problems Test 2: p = 0.71; Test 2 + Test 3; p = 0.87; C-hat = 0.98). These results show the data to be neither over- nor under-dispersed, thus deeming additional corrections within the robust design unnecessary. According to AICc, the most parsimonious model had constant survival probability, random emigration probability (not time-dependent) and a capture probability varying between and within primary periods (Table 3). The model accounting for heterogeneity with 2 mixtures had little or no support (model 45).

**Table 3:** Robust Design candidate models for survival probability (s), capture probability (p), temporary emigration probability (γ) and abundance (N).

Model nr in Program Mark	Model	AICc	Δ AICc	AICc Weights	Deviance	No parameters
1	s(.) γ“(.) = γ’(.) p(t*s) N(t)	768.4	0.00	0.40	1349.1	73
2	s(annual) γ" = γ’ = 0 p(t*s) N(t)	770.3	1.87	0.16	1351.0	73
3	s(.)γ “(.) γ’(.) p(t*s) N(t)	770.7	2.29	0.13	1348.3	74
4	s(.) γ" = γ’ = 0 p(t*s) N(t)	770.9	2.43	0.12	1354.6	72
5	s(.) γ"(annual) = γ’(annual) p(t*s) N(t)	771.6	3.11	0.08	1346.0	75
6	s(annual) γ"(.) = γ’(.) p(t*s) N(t)	772.0	3.57	0.07	1346.5	75
7	s(.) γ"(annual)γ’(.) p(t*s) N(t)	774.6	6.17	0.02	1345.9	76
8	s(seasonal) γ"(.) = γ’(.) p(t*s) N(t)	776.6	8.13	0.01	1347.9	76
9	s(.) γ"(season) = γ’(season) p(t*s) N(t)	776.7	8.29	0.01	1348.1	76
10	s(.) γ"(.) γ’(season) p(t*s) N(t)	778.2	9.80	0.00	1346.4	77
11	s(season) γ" = γ’ = 0 p(t*s) N(t)	778.7	1.02	0.00	1353.1	75
34	s(.) γ" = γ’ = 0 p(s) N(t)	927.4	158.99	0.00	1635.6	25
43	s(.) γ" = γ’ = 0 p(t) N(t)	990.5	222.05	0.00	1698.6	25
45	s(.) γ" = γ’ = 0 pi(t) p(t)	990.5	222.02	0.00	1698.6	25

Models are ranked by their AICc values. Δ AICc is the difference in the AICc of a model from that of the minimum AICc model. AICc weight indicates the support of the selected model over the others. Deviance is a measure of model fit. At all times, recapture probability (c) was set equal to capture probability (p) and is therefore not mentioned. Notations: (.) constant, (t) time-dependence between primary periods, (s) time-dependence within primary periods, (γ") probability of temporal emigration, (γ’) probability of remaining outside the study area, (γ" = γ’ = 0) no emigration, (γ" = γ’) random emigration, (γ" γ’) Markovian emigration.

Although all the models with no emigration were rejected in the LRT in favour of models with migration (Random: χ² = 5.49, p < 0.05; Markovian: χ² = 6.28, p < 0.05), the model with Markovian emigration could not be rejected in favour of a random emigration (χ² = 0.79, p = 0.37). None of the models with annual, seasonal and full time dependence of γ could be rejected (annual: χ² = 3.07, p = 0.21; seasonal: χ² = 1.01, p = 0.79; full time dependence: χ² = 9.35, p = 0.406). The constant survival probability was not favoured in the LRT when compared to annual, seasonal and full time dependent variation (annual: χ² = 2.61, p = 0.28; seasonal: χ² = 1.18, p = 0.76; full time dependence: χ² = 5.45, p = 0.86). Time-dependence of capture probability between and within primary periods did contribute significantly to the model fitting (between primary periods; χ² = 344.06, p < 0.01; within primary periods: χ² = 281.03, p < 0.01).

### Adult survival probability and temporary emigration

Adult survival probability was very similar in all the candidate models, and the resulting average survival rate (weighted over the best fitting models) varied between 0.97 (± 0.037 SE) and 0.99 (± 0.010 SE). The probability of temporal emigration was equal to the probability of remaining outside the area (γ” = γ’), and averaged 0.047 (95%CI = 0.004–0.637) over the models. The derived return rate of temporary emigrants (1 - γ’) was 0.953, equal to the probability of remaining in the area (1 - γ”). Capture probability varied between 0.02 and 0.66.

### Abundance estimates

**Fig 2:**
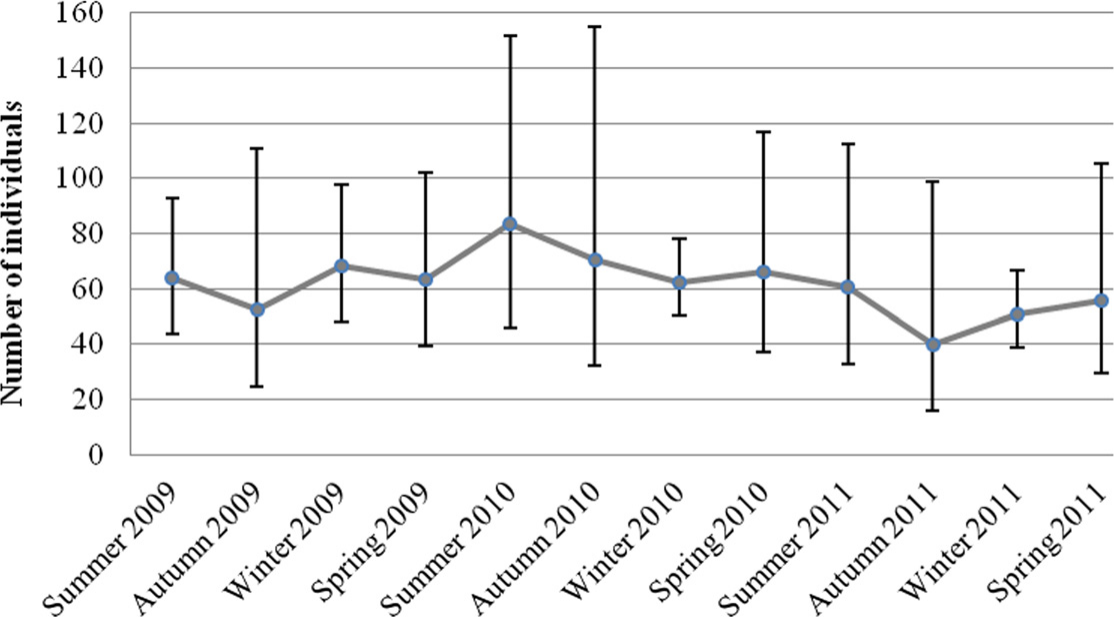
Total abundance estimates with 95%CI for the study area of 2009, 2010 and 2011, corrected for the proportion of unmarked individuals in the population.

The total abundance of dolphins in the study area, corrected for unmarked individuals, varied between 40 individuals (95%CI = 16.1–98.8) and 83 individuals (95%CI = 45.8–151.8) (Fig. 2; Table 4). The proportion of marked individuals in the population averaged 0.65 (± 0.05 SD).

**Table 4:** Seasonal abundance estimates of bottlenose dolphins in the study area.

		N_marked_	SE	Proportion of marked dolphins	N_total_	SE
2009	Summer	40	5.8	0.6300B00310.09	64	12.3
	Autumn	38	13.9	0.73±0.14	52	20.9
	Winter	44	6.1	0.64±0.08	68	12.5
	Spring	44	9.3	0.69±0.03	63	15.6
2010	Summer	52	13.9	0.62±0.07	83	26.1
	Autumn	40	14.0	0.57±0.09	71	29.5
	Winter	39	1.0	0.63±0.12	63	7.1
	Spring	42	10.2	0.63±0.05	66	19.6
2011	Summer	36	10.0	0.60±0.08	61	19.6
	Autumn	28	12.6	0.71±0.09	40	19.5
	Winter	35	3.1	0.69±0.09	51	7.0
	Spring	36	1.9	0.65±0.05	56	18.6

N: abundance estimate of marked individuals, SE: standard error, Proportion of marked dolphins: seasonal average (±SD) of the proportion of marked individuals in the dolphin groups encountered, N_total_: total abundance estimate (marked and unmarked individuals).

### Birth demographics

Between 2006 and 2011, a total of 25 different calves was photographed closely associated with 14 identifiable females of reproductive age in the population, 21 of which were born after 2006. The remaining 4 calves were already born when this study commenced. Over the study years, 4 calves disappeared abruptly at an age < 2 yrs suggesting that they had died. In total, 14 calves are known to have survived the first 3 years of life, of which at least five could be included into the photo-identification catalogue due to their first distinct marks and scars. By the end of 2012, 7 calves were still present in close association with their mother and were thus assumed to be still at pre-weaning age. When these dependent calves are excluded from calculation, it can be concluded that 14 out of the 18 calves (78% i.e., 25 calves minus 7 pre-weaning calves) of known age, survived to post-weaning age.

The birth season could be determined for 18 of the 25 calves, of which 83% (n = 15) were born in late spring/early summer. During the study period, the average calving interval equalled 3.5 ± 1.03 years (n = 14), ranging from 2 to 5 years, with an estimated 3.5 births/year (21 births in 6 years) in the entire population. Accounting for a maximum estimated population size of 83 individuals, this results a minimum annual birth rate of 4.2%.

### Population viability analysis

Although most parameters needed for the population viability analysis were available through this study, some life history parameters were obtained from two long-term studies in Sarasota Bay [46] and Bay of Island [47]. These included age of first reproduction (set at 10 and 11 for females and males, respectively), sex ratio (50:50), maximum life-span (50 yrs) and the proportion of males in the breeding pool (75%). In accordance to the results of this study, population size was set at 83 individuals, mortality of calves aged 0–3 was set to 22%, whereas mortality of individuals > 3 years old was set to 2%. At this stage, the proportion of adult females breeding per year was set at 25% [(3.5 births/yr) / 14 reproducing females]. The carrying capacity was set on twice the current population size, resulting in 166 individuals [45]. Simulations were run for 100 yrs, and each scenario was repeated 500 times [45,48]. Under the initial scenario, the local population is assumed to increase at a rate of 1.4% per year.

A sensitivity analysis was subsequently performed, in which each parameter was varied and the consequential change to the rate of population increase and time to local extinction was recorded. This analysis indicated that calf mortality had the greatest impact on the population growth rate: the population was estimated to decrease by 2.2% per year if calf mortality (between 0–3 years of age) was doubled to 44% (resulting in a mean local functional extinction time of 77 years). Furthermore, the proportion of reproducing females per year had a significant impact on the population trend. When reduced to half of the initial scenario (i.e. 12.5%), the population was estimated to decrease by 1.1% per year resulting in a mean local functional extinction time of 92 years. When only the female adult mortality was doubled compared to the initial scenario, the population was expected to decrease by 0.03% per year. On the other hand, a similar increase of male adult mortality did not have a significant effect on the population growth rate, nor did any of the other parameters.

## Discussion

### Adult survival probability

The estimate of apparent adult survival is slightly higher than those reported in other regions [9,12,49,50,51,52]. Although the best fitting model suggests a constant survival rate, models accounting for time dependency of survival rates never dropped below 0.93 (± 0.048 SE).

### Birth demographics

The calving interval of 3.5 ± 1.03 years appears to fall into the normal range for bottlenose dolphins [43,53,54]. However, the minimum annual birth rate was estimated to be somewhat lower than values reported for other bottlenose dolphin populations [20,43,49,54]. Consistent with the reported low birth rate, the average proportion of unmarked (i.e. mostly immature) individuals in the population was considerably lower in the study period (0.35) compared to 2008 (0.47) [3], most likely due to recruitment of calves and their acquisition of permanent scars at a faster rate than the addition of new calves into the population.

Considering a normal calving interval, the low minimum annual birth rate should be related to a low number of successfully reproducing females in the entire population rather than to a low number of calves born from the known reproducing females. As such, considering a photo-identification catalogue containing minimum 57 adults and supposing a 1:1 ratio of males vs. females at birth, twice as many than the reported 14 reproducing females would be expected within this population. The small proportion of documented calves dying before being weaned from their mothers (22%) suggests a low calf mortality [49,53]. However, as only 38% of the calves born after 2006 were documented with foetal folds, it is likely calves were born and lost before being documented, suggesting birth rate, calf mortality and possibly number of reproductive females are underestimated.

Values suggest the recruitment rate of calves appears to be insufficient to support the size of the population: the recorded calf mortality amounts to 0.7 animals / year (4 calves in 6 years), which represents a minimum annual mortality rate of 1% of the registered population or a third of the total estimated mortality. At an estimated adult mortality rate of 2%, a population growth of 1.2% per year is possible only when ignoring the mortality of immature dolphins (considering the minimum annual birth rate of 4.2%). If their mortality rate is equivalent to that of adults or higher, this population is declining. Accordingly, the sensitivity analysis of the PVA indicated a significant population decline when assuming a smaller proportion of reproducing females in the population (due to many non-reproducing adult females) or a higher calf mortality (due to an underestimated level of unsuccessful reproduction).

A likely underestimated calf mortality and thus the unsuccessful reproduction in certain females might be caused by a lack of experience in some females as well as predation or other factors reducing the reproductive success of female bottlenose dolphins [53,55,56]. The predation pressure in the study area is suggested to be low according to the lack of visual predatory scars from shark or killer whale bite marks. Limited toxicological research in Argentina, however, indicated elevated levels of heavy metals in bottlenose dolphins in various regions along the Argentinean coast [57,58]. Within our study area, research on the accumulation of heavy metals in crustaceans (*Chasmagnathus granulate*), molluscs (*Brachydontes rodriguezi*), sea lions (*Otario flavescens*) and even in children living near the study area revealed elevated levels of lead, copper, zinc and cadmium in their systems, assumed to be related to a former mining activity in the region that left waste piles still leaching various metals into the environment more than two decades after closure of the mines [59,60,61,62]. Claps [63] states “the high levels of accumulation in mussels of lead, zinc, copper and cadmium in the bay of San Antonio might pose a contamination risk throughout the food chain, proving a great threat to larger predators”. Frodello et al. [64] indicated that metal pollutants pass from the tissue to the milk in lactating bottlenose dolphins. Lead is also known to pass the placenta affecting the nervous system of the foetus [65]. Contaminants passed down from the mother as well as other health related stresses may compromise the immune response of new-born dolphins [66], with consequently primiparous females having an increased risk of reproductive failure [67]. We suggest the reported levels of pollution should be regarded as a major concern for the health and reproduction of the bottlenose dolphins residing in the region.

### Population at risk?

This population of bottlenose dolphins is relatively small and likely to be declining. The reported high contamination with heavy metals [59,68] as well as possible overfishing of the area [69] are suspected to be among the causes of this decline and need to be investigated further. A genetic study conducted by Fruet et al. [70] indicated the population to be an “evolutionary significant unit” within the south-western Atlantic, stressing their apparent genetic isolation. The low genetic diversity (comprising only one haplotype) found by the same study further supports our findings of a small (remnant) population. Our results also suggest that this population is highly vulnerable and at risk, because cetacean populations of less than 100 individuals are known to have higher extinction risks due to stochastic events [71,72,73,74]. Therefore, the toxicological burden of the population should be monitored, and nationwide measures need to be taken to protect this species and its habitat, including a controlled management of rural, urban and industrial wastes and run-offs, protective laws to limit harassment, as well as educational projects to increase public awareness. Considering that the species has nearly disappeared from the regions north and south of the study area [1,2], continuous failure in their conservation could have a devastating effect on the presence of coastal bottlenose dolphins in this South-American country.

### A common species?

The coastal lifestyle and site-fidelity of coastal bottlenose dolphins may have obfuscated the need for more extensive research and conservation efforts in Argentina in former years. We believe that this misconception about the status of coastal bottlenose dolphin populations might also be wide-spread on an international scale, exacerbated by a global attitude towards the *Tursiops* species. As human urbanizations increase along coastlines, coastal bottlenose dolphins are particularly vulnerable to ensuing anthropogenic impacts [75]. Additionally, research increasingly indicates coastal bottlenose dolphin populations are more isolated than previously believed, a finding that makes them even more vulnerable. To test our assumption, we reviewed the available literature and found an ever increasing number of coastal bottlenose dolphin populations worldwide have been reported to be vulnerable or declining (Table 5). Not a single population, however, was described to be increasing. Therefore we suggest that the misconception about the global wellbeing of coastal bottlenose dolphin needs to be re-evaluated while leaving behind the “one species, one assessment” approach for a more fine-scale approach based on improved scientific collaboration.

**Table 5:** List of regional bottlenose dolphin populations (*Tursiops sp*.) reported to be declining or vulnerable (defined as containing fewer than 1000 mature individuals [76]).

**Region**	**Population**	**Population size**	**Population trend**	**Possible threats**	**Reference**
Europe	Moray Firth, Scotland, UK	~130	- 5% pa	Pipeline construction, dumping of dredge spoils, commercial fishing, dolphin-watching	[20,73,77,78]
	Sound of Barra, Scotland, UK	~15	n/a	Fishing industry, gas- and oil-related activities, coastal developments	[79]
	Cornwall, SW England, UK	~30	n/a	Bycatch, disease, prey depletion	[89,81,82]
	Cardigan Bay, UK	~200	n/a	Boat traffic	[83,84,85,86]
	Shannon Estuary, Ireland	113	n/a	Pollution, habitat degradation, bycatch, dolphin watching	[87,88,89]
	Channel Islands, France	66	n/a	n/a	[90]
	Molène Archipelago, Brittany, France	~50	n/a	n/a	[86]
	Ile de Sein, France	~20	n/a	n/a	[86]
	Southern Galicia, Spain	123–664	n/a	Fishing industry, contamination	[91,92,93]
	Sado Estuary, Portugal	~25	n/a	Habitat degradation	[94,95]
	Asinara Island National Park, Italy	22	n/a	Interaction with fisheries	[96]
	Lampedusa Island, Italy	249–446	n/a	Interaction with fisheries, boat traffic	[97,98,99]
	Gulf of Trieste, Slovenia	47	n/a	Contamination, recreational boats, fishing industry, habitat degradation	[100]
	Kvarneric, Croatia	~200	-50% in past 50 ys	Historical killing, habitat degradation, nautical tourism, fishing activities	[101,102,103]
	Ionian Sea, Greece	48	n/a	Overfishing	[10]
	Amvrakikos Gulf, Greece	148	n/a	Contamination, habitat degradation, overfishing	[8]
	Israeli Mediterranean Sea, Israel	85	n/a	Fishing industry	[104]
	Kerch Strait, Black sea	127	n/a	Noise pollution, habitat degradation	[6,105]
Australasia	Fiordland, New Zealand (3 subpopulations)	205	- 2.8% pa (Doubtful Sound)	Freshwater discharge, dolphin-watching	[7,106,107,108]
	Bay of Island, New Zealand	483	- 5.8–7.5% pa	Dolphin-watching	[52]
	Hauraki Gulf, New Zealand	162	n/a	Shipping traffic	[109]
	Marlborough Sounds, New Zealand	195–232	n/a	Recreational vessel traffic, ecotourism, aquaculture, contamination from runoffs	[110]
	Moreton Bay, Queensland, Australia	446+193 (North+South)	n/a	Urban development	[111]
	Port Stephens, NSW, Australia	~160	n/a	Dolphin watching, contamination	[112,113]
	Jervis Bay, NSW, Australia	108	n/a	Dolphin watching, contamination	[112]
	Clarence River Estuary, NSW, Australia	71	n/a	Fishing activities	[114]
	Richmond River Estuary, NWS, Australia	34	n/a	Fishing activities	[114]
	Useless Loop, Shark Bay, WA, Australia	~208	n/a	Dolphin based tourism, habitat degradation	[9,115]
	Bunburry, WA, Australia	139	n/a	Contamination, prey depletion	[116]
	Pilbara, WA, Australia	n/a	n/a	Commercial trawl fishery	[117]
	Mirura Island, Japan	~220	n/a	Dolphin based tourism	[54]
Africa	Kwazulu-Natal, South Africa	~ 900	n/a	By-catch in shark nets	[118,119,120]
	West Africa	n/a	n/a	Incidental and directed takes	[121]
	Zanzibar, Tanzania	136–179	n/a	Historic hunting, bycatch, dolphin watching,	[39]
	São Tomé Island, Democratic Republic of São Tomé and Príncipe	37	n/a	(Illegal) Fishing activities	[122]
Central America	Bocas del Toro, Panama	~150	n/a	Dolphin watching	[123]
	Drowned Cayes, Belize	122	n/a	Overfishing, contamination	[124]
	Turneffe Atoll, Belize	~86	n/a	Tourism, fishing activities	[125]
	Tamiahua, Mexico	177	n/a	Artesanal fishing	[126]
	Tuxpan, Mexico	161	n/a	Artesanal fishing	[126]
	Coast of Tabasco, Mexico	300–573	n/a	n/a	[127]
South America	Margarita Island and Los Frailes Archipelago, Venezuela	<60	n/a	Directed catches, tourism	[128]
	Gulf of Guayaquil, Ecuador	115	n/a	Bycatch	[129,130]
	Chañaral, Damas, Choros and Gaviota Islands, Chile	30–35	n/a	Dolphin based tourism, bycatch	[131,132,133]
	Cagarras Archipelago, Brazil	n/a	n/a	Fishing activities, marine traffic, contamination	[134,135]
	Mirim, Imaruí and St. Antônio Lagoons, Brazil	~54	n/a	Incidental catch, contamination	[12,136]
	Patos Lagoon Estuary, Brazil	~84	n/a	Incidental catch, collisions with fishing boats	[137,138,139]
	Coast of Uruguay	~55	n/a	Overfishing, habitat degradation, incidental catch	[140]
	Buenos Aires, Argentina	n/a	n/a	Overfishing, habitat degradation	[1]
	Peninsula Valdes, Argentina	n/a	n/a	Overfishing, habitat degradation	[2]
	Bahia San Antonio, Argentina	83	n/a	Contamination	This study
